# Estimation of Water Depth on Road Surfaces Using Accelerometric Signals

**DOI:** 10.3390/s22228940

**Published:** 2022-11-18

**Authors:** Ebrahim Riahi, Wiyao Edjeou, Sébastien Buisson, Manuela Gennesseaux, Minh-Tan Do

**Affiliations:** 1 TS2-LMA, Univ Gustave Eiffel, IFSTTAR, F-13300 Salon de Provence, France; 2 AME-EASE, Univ Gustave Eiffel, IFSTTAR, F-44344 Bouguenais, France

**Keywords:** road safety, water depth, accelerometric signal, skid resistance, signal filtering

## Abstract

The paper presents an experimental study conducted to evaluate the feasibility of using accelerometers as an indirect means to estimate water depths on road surfaces. It makes use of the vibration of the vehicle’s wheel arch due to water droplets projected by a tire rolling on a wet road surface. A trailer equipped with a wheel and towed by a van was used. The test setups to spread water on the road surface and before the test wheel, measure the water depth and visualize the water spray are described. The test program, conducted on a test track closed to the traffic, includes three surfaces and two speeds. Visualization of water flows by means of high-speed cameras makes it possible to choose a suitable location for the accelerometers. It turns out that signals provided by the accelerometers are affected by the trailer’s movement; a filtering method has been successfully developed to remove noises. Results show a tight relationship between the mean amplitude of accelerometric signals and actual water depths. Discussions are made in terms of effects of the vehicle speed and the road surface texture. Perspectives for using the developed system to improve passenger safety under autonomous driving conditions are presented.

## 1. Introduction

It is well known that the presence of water on a road surface affects drivers’ safety. While many studies have been dedicated to the link between precipitation, even at light intensity, and vehicle crashes [1,2,3,4], statistical analyses [4,5,6,7] also show that the accident risk is still high after a rainfall. The higher accident risk on wet roads depends on two concurrent factors: the reduction of the road surface skid resistance and the drivers’ perception.

Skid resistance describes the contribution that the road makes to tire–road friction. Friction forces are generated by the contact between the tire rubber and the road surface asperities. In the presence of water at the tire–road interface, part of this contact is supported by the water film, and therefore the coefficient of friction is reduced [8]. Experimental evidence and numerical simulations [9,10,11,12,13,14] show that the coefficient of friction decreases in an exponential way when the water depth increases. The friction drop is further aggravated if the vehicle speed increases due to a reduced time to drain water from the tire–road interface [8]. Results from Veith [9] for tests performed with passenger cars with speeds varying from about 30 km/h to 90 km/h and water depth varying from about 0.1 mm to 5 mm show, on a friction-logarithm (water depth) graph, lines with negative slopes which increase for increasing speed; it means that increase of road wetness induces more deterioration of skid resistance at higher speed.

Currently, no information related to the reduction of skid resistance exists in the vehicle, and drivers still react from their experience, mainly from their perception of the road wetness. Flooded roads or heavy precipitation can induce a sense of insecurity that incites drivers to reduce the vehicle speed. For conditions which do not alter the visibility, such as after a rainfall or under a drizzle, drivers can maintain a speed as high as on a dry road. Yet, as the presence of a thin water film (less than a tenth of a millimeter) would be enough to significantly reduce tire–road friction [8,12], maintaining a high speed on a wet road increases the accident risk. This explanation corroborates the high accident risk during light rain [4,6].

Based on the explanations above, it can be said that water is the factor triggering the risk of crash on wet road due to unsuitable vehicle speed. There is then a need to detect the presence of water on a road surface and quantify the water depth. In the context of driver assistance—and, by extension, autonomous driving—this information is primordial because, combined with the characteristics of other influencing factors (road surface, tires, etc.), it helps to estimate the actual skid resistance and, in turn, warn the driver or automatically adapt the vehicle speed.

**Figure 1:**
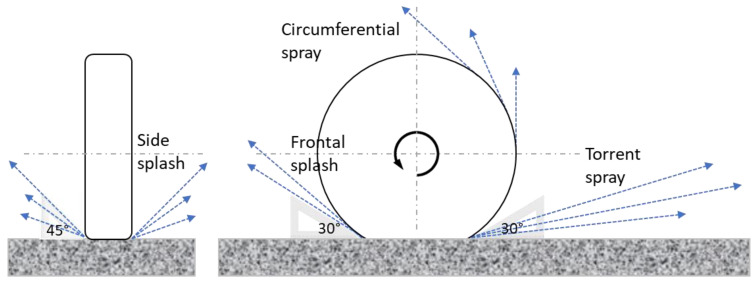
Flows generated by the ejection of water from the tire–road contact area (left: front view of the tire; right: side view of the tire; the direction is from the right to the left).

Sensors exist to measure the thickness of water films on a road surface [15]. However, commercially available sensors cannot be mounted on a fleet of passenger cars due to their dimensions and their costs. Among existing technologies (cameras, radars, etc.) developed to make a system more suitable for mass production, as presented thoroughly by Schmiedel et al. [16] and Döring et al. [17], the present paper focuses on the measurement of vibrations of a vehicle’s wheel arch due to water spray. According to Weir et al. [18], there are four mechanisms related to water spray: bow, side splash waves, tread pickup and capillary adhesion. Schmiedel et al. [19] reformulated these mechanisms as frontal splash, side splash, torrent spray and circumferential spray, respectively; these terms are illustrated in Figure 1 (Section 2) and will be used in the rest of the paper. The first use of water spray as an indirect measurement of water depth has been found in works published by Prevost and coauthors (2012). These authors used accelerometers to measure vibrations of the wheel arch due to droplets projected by the right front tire of the vehicle. Based on extensive tests (5 surfaces, 5 speeds, 8 types of tires), an indicator linked to the accelerometer signal (definition not provided by the authors) has been found to be well correlated to the water depth (lower than 0.25 mm) measured by an optical sensor [15] in the same wheel path. In the recent works of Schmiedel et al. [19,20], efforts have been made to assess the effect of the sensor mounting (on the backside of the wheel arch liner, on the side skirt, at the car’s underbody) on the detection of different water flows. The water depths simulated in Schmiedel’s experiments are higher than those in Prevost’s work (generally between 0.5 and 1 mm and, at the extreme case, up to 2 cm). In Schmiedel at al. [16], these authors report the following: the circumferential spray is related to low water film thickness (less than 0.5 mm); above 0.5 mm of water depth and at low speed (40 km/h), the torrent spray can be observed; and above 0.5 mm of water depth and at high speed (up to 120 km/h), the side splash can be seen in parallel to the onset of hydroplaning. The most recent work dealing with water spray is presented in Döring et al. [17]. Instead of accelerometers, these authors have used capacitive transducers arranged as an array mounted on the backside of the right front wheel arch. The location of the developed system aims at capturing the circumferential spray and detecting water depths lower than 0.5 mm. Depending on water depth values, eight wetness levels–dry, damp, wet and very wet with sublevels—have been defined. Experiments conducted with a passenger car at speeds varying from 15 km/h to 50 km/h on a straight asphalt section have shown conclusive results in terms of correspondence between the wetness levels determined by a commercial water sensor and those determined by the transducers.

The works presented above show that estimating the road wetness from water spray is a promising approach. This approach presents a significant advantage in terms of risk detection because data can be collected while the vehicle is moving without necessitating any special maneuver (like braking); therefore, the risk can be known before the driver perceives it. In this paper, the approach based on accelerometers is further investigated. The objective of the work presented hereafter is to address the following questions that remain open:

1. Where is the appropriate location for the accelerometers on a wheel arch?
2. How can accelerometric signals be processed to extract the relevant information?
3. What is the relationship between accelerometric signals and actual water depths?

In the following sections, the research methodology is first presented; the experimental program, with a description of the sensors, the test setup and the test program, is then provided; and finally, results are presented and discussions are made with respect to the mentioned questions.

## 2. Methodology

The four water flows while a car wheel rolls over a wet surface are illustrated in Figure 1. The names of the flows are those employed by Schmiedel et al. [19]. In this work, focus is made on the estimation of water depths lower than 1 mm. These water depths correspond to water accumulated on the road surface during a fine rain (rainfall intensity of 5 mm/h) [21], which represents a critical driving condition because the driver cannot perceive the presence of water on the surface.

Based on findings of Schmiedel et al. [16], related to the relationship between road wetness and water sprays, focus is placed on the effect of circumferential and torrent sprays (low water depth and low vehicle speed). The study involves three key steps:

1. In the first step, efforts are made to visualize water sprays. High-speed cameras are used for this purpose. Videos are used to better understand the distribution of water droplets and, depending on the spray intensity, choose appropriate locations for the accelerometers on the wheel arch.
2. In the second step, signals are processed to extract relevant information with respect to road wetness.
3. In the third step, correlation is established between indicators calculated from accelerometers’ signals and water depths.

Within the frame of this study, it was decided to conduct tests on a trailer equipped with a wheel and towed by a van. This configuration constituted a preliminary step (before moving toward a passenger car) because the dynamics of the trailer used are similar to a car wheel suspension system (quarter of a passenger car), and it was possible to control the quantity of water before the test wheel and visualize the water flows.

Tests were conducted on the test track of Gustave Eiffel University in Nantes, which is closed to traffic and possesses surfaces covering a wide range of textures. The facility was then suitable because, beside the possibility to conduct calibration tests during the development of the system, the effect of surface texture and vehicle speed on the results could be studied there.

## 3. Experimental Program

In this section, a description of the sensors is first given. The setup developed, using accelerometers, to capture water sprays is then detailed. The test program is finally presented.

### 3.1. Sensors

#### 3.1.1. Accelerometers

**Figure 2:**
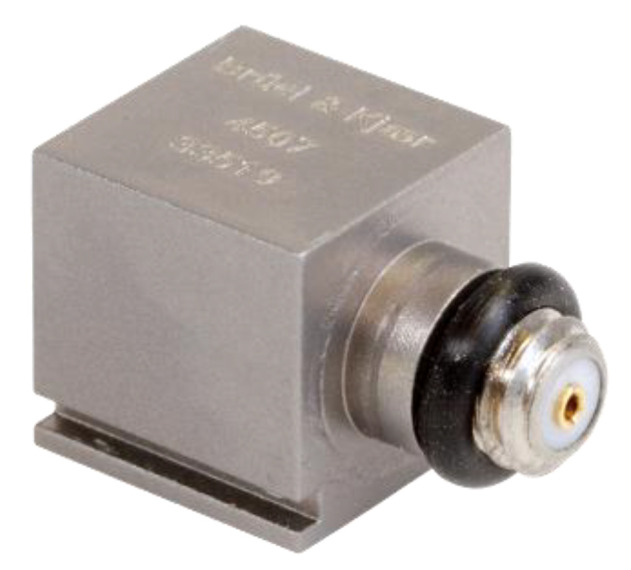
Brüel & Kjær piezoelectric accelerometers type 4507.

To measure the impact of water droplets on the wheel arch liner, Brüel & Kjær piezoelectric accelerometers (type 4507) were used (Figure 2). These accelerometers were chosen because of their use—and thus proven performance—in previous studies [19,22] and their small dimensions (and low weight: less than 5 g). More technical data of these sensors can be found online at the following link: https://www.bksv.com/es/transducers/vibration/accelerometers/ccld-iepe/4507-b (accessed on 16 November 2022).

The accelerometers were connected to the data acquisition system (see Section 3.2.2). Measurements were performed with a sampling rate of 38 kHz.

#### 3.1.2. High-Speed Camera

**Figure 3:**
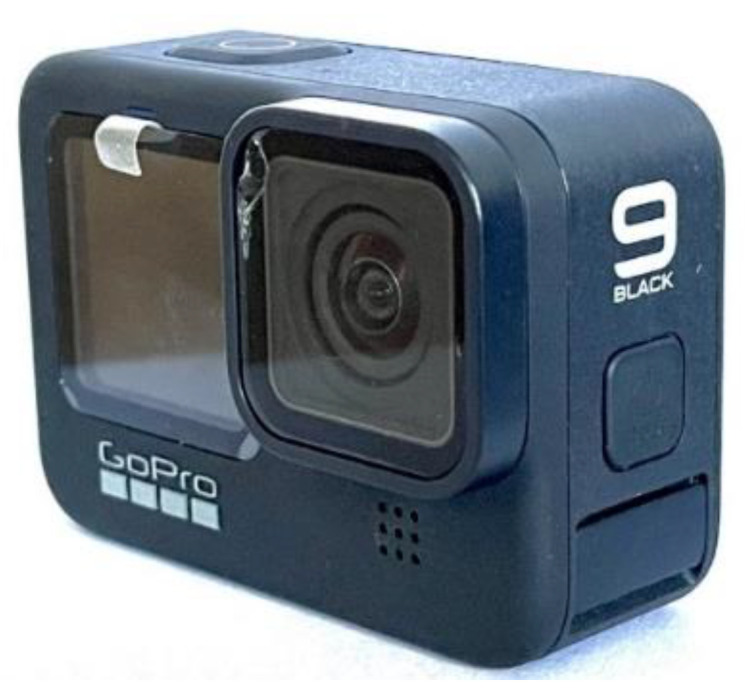
GoPro camera (example shown for a HERO9 model).

Water droplets were visualized by means of three GoPro cameras (Figure 3) located at 3 different locations (see Section 3.2). HERO5, HERO7 and HERO9 models were used to capture the torrent and circumferential sprays. The video frame rate was 120 images per second with a resolution of 1080p.

#### 3.1.3. Noncontact Sensor to Measure Water Depths

**Figure 4:**
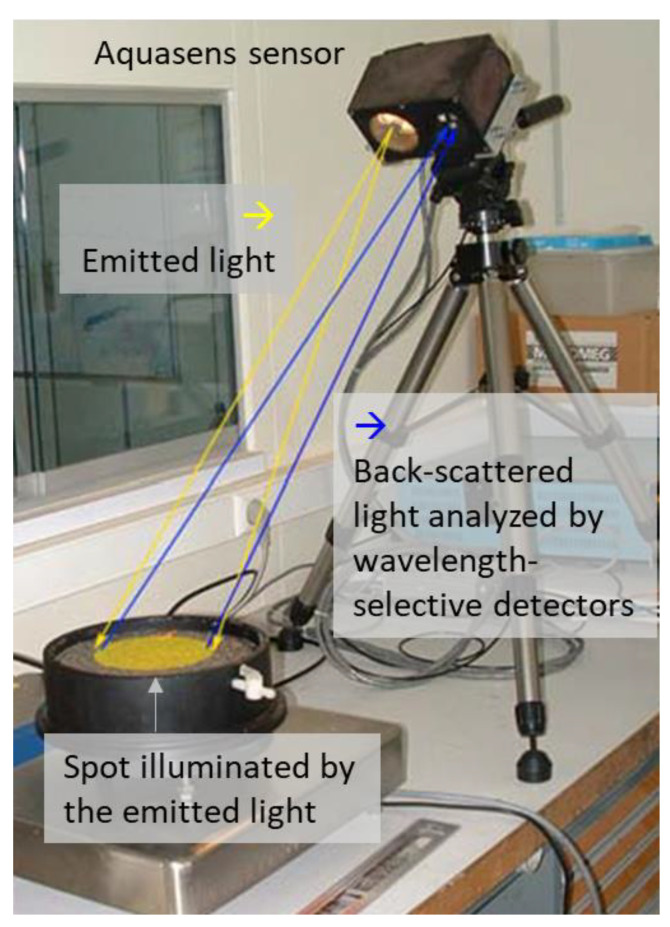
Aquasens sensor (configuration used in laboratory to calibrate the sensor) and illustration of the operating principle.

Measurements of the water depth were performed by the so-called Aquasens sensor (Holzwarth Mechatronik, Adelmannsfelden, Germany) developed by Holzwarth et al. [15]. The operating principle (Figure 4) is based on absorption properties of water for radiations located near the infrared wavelength region. The device is equipped with a light source and receivers. The road is illuminated with white light which is altered by the water on the road surface and then reflected to the receivers. The reflected ray is then analyzed to determine the water depth. More details can be found in Holzwarth et al. [15].

**Figure 5:**
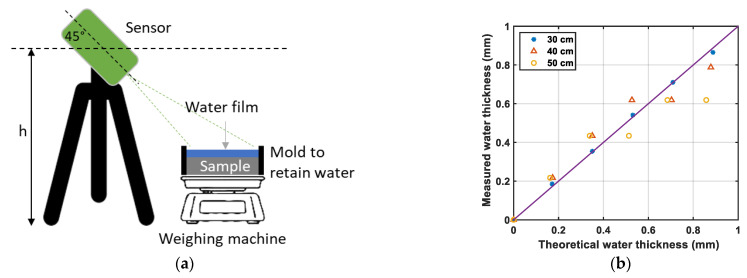
Comparison of water depths measured by the Aquasens at different heights h ((**a**) setup; (**b**) results).

This device has the advantage of continuously measuring the water thickness while the vehicle is moving with a high sampling frequency (here 38 kHz). The device should be placed at a recommended height of 40 cm from the test surface and inclined by an angle of 45° toward the road surface (Figure 5). Two sensitivity ranges of 0–1 mm and 0–10 mm, respectively, can be used for water thickness measurement. With respect to the targeted water depths of this study (lower than 1 mm), only the range of 0–1 mm was used.

Tests were first performed in the laboratory in static mode (Figure 5) to compare water depths measured by Aquasens and estimated from weighing. Circular samples of road surface with a diameter of 225 mm were placed inside a waterproof mold. The sample and the mold were weighed first when the surface was dry and then after different water quantities were added on the sample surface; the difference in weight provided the volume of added water and, by dividing it by the surface of the sample, the mean water depth.

Tests were also conducted for different heights (h) to assess the effect of this factor on the measurements. The comparisons between the measured and estimated water thickness for heights of 30, 40 and 50 cm are presented in Figure 5b. It can be seen that measured and estimated water thickness are very closed, mainly for a height of h = 30 cm.

### 3.2. Test Setup

**Figure 6:**
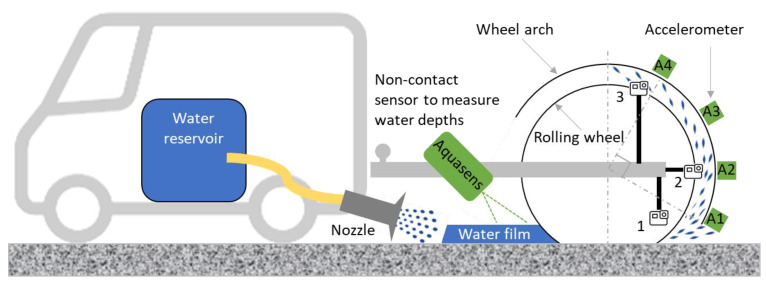
Illustration of the test setup (dimensions not respected; letters A1 to A4 indicate the location of accelerometers; numbers indicate the location of GoPro cameras).

The test setup is illustrated in Figure 6. A trailer equipped with a wheel and towed by a van was used to facilitate preliminary tests, which aimed at better understanding water sprays’ mechanisms and defining appropriate locations for the accelerometers. The measuring wheel, loaded at 2500 N, was simply rolling on the test surface at the same speed as the towing van’s speed. The test tire was a Michelin tire 185/60 R15 inflated at 0.22 MPa.

Different parts of the setup are commented on in the following sections.

#### 3.2.1. Setup to Spread a Water Film on the Test Surface

**Figure 7:**
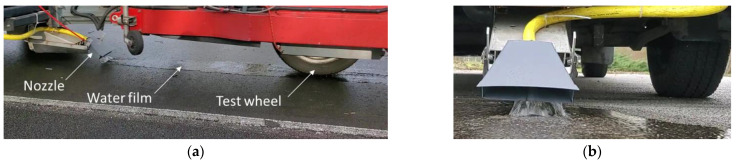
(**a**) Water film on the test section; (**b**) Nozzle (close view on the right photo) to wet evenly the surface.

To wet the wheel path evenly, an on-board wetting system was developed. Water from a reservoir (1 m^3^) inside the van flowed by gravity and was directed toward the wheel path by means of a nozzle equipped with a head which distributed the water over a width of 25 cm (Figure 7).

The Aquasens was mounted on the trailer to measure water thickness at 20 cm in front of the measuring wheel (Figure 6). The sensor was placed 30 cm from the ground with an inclination angle of 45°.

#### 3.2.2. Setup to Measure the Impact of Water Droplets

**Figure 8:**
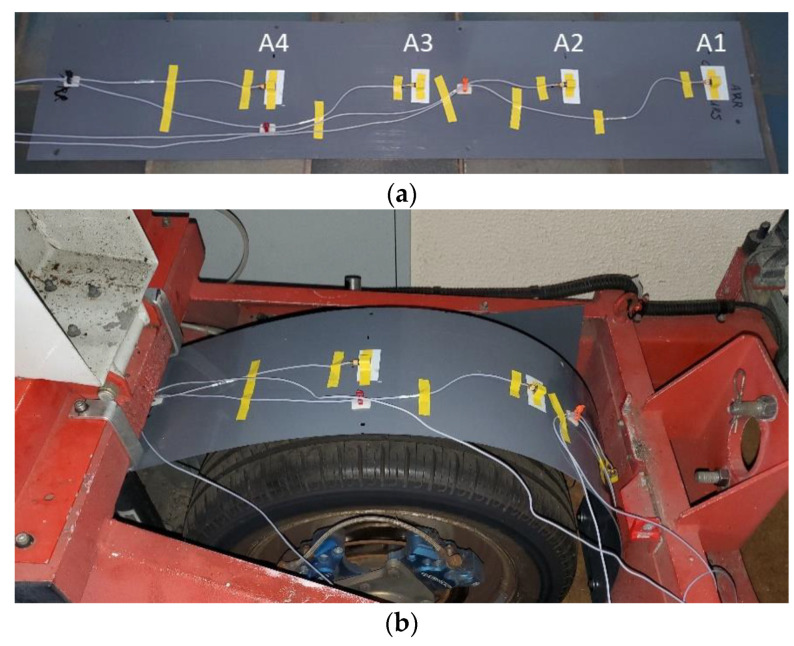
(**a**) The PVC plate used to reproduce a wheel arch and the location of the accelerometers; (**b**) the wheel arch and the accelerometers mounted on the trailer.

A wheel arch liner was made from a PVC plate for the measuring wheel. The accelerometers were fixed behind—with respect to the side receiving water droplets—the wheel arch liner using double-sided adhesive tape at four different positions (Figure 8; see also Figure 6 for the location of the accelerometers once the wheel arch was mounted on the trailer). The first accelerometer (A1) was located at the bottom of the wheel-arch liner, and the following ones were spaced every 30° (Figure 6).

For data acquisition, the accelerometers and Aquasens were connected to a HBM QuantumX data acquisition system. Catman software was used to set the data acquisition parameters.

#### 3.2.3. Setup to Visualize Water Droplets

The location of the three GoPro^®^ cameras is shown in Figure 6. The first camera (HERO5) was installed at the tire-road contact level to visualize the torrent spray. The second camera (HERO7) was fixed behind the wheel at the same location as the accelerometer A2. The third camera (HERO9) was located at the top of the wheel.

### 3.3. Test Program

Tests were conducted at two speeds (30 and 50 km/h) on three road surfaces. Each configuration (surface, speed) was repeated three times. The test sections were part of the test track of Gustave Eiffel University (campus of Nantes). The first surface named C1 is a surface dressing with a low macrotexture (MTD = 0.38 mm). The second surface named E1 is a semicoarse asphalt concrete with a maximum aggregate size of 10 mm and MTD value of 0.95 mm. The third surface named M3 is a very thin asphalt concrete with a maximum aggregate size of 4 mm and an MTD value of 0.85 mm. Photos of these surfaces are shown in Table 1.

MTD, acronym of the mean texture depth, is a parameter characterizing the road surface macrotexture, which corresponds to surface irregularities whose dimensions range between 0.1 mm and 20 mm vertically, and between 0.5 mm and 50 mm horizontally (ISO, 1997). The MTD of a surface is obtained by dividing a known volume of glass beads by the surface of a circular patch formed by spreading the glass bead over the surface; details of the test protocol can be found in standard ISO 13473-1 [23].

## 4. Results

### 4.1. Visualization of Water Sprays

**Figure 9:**
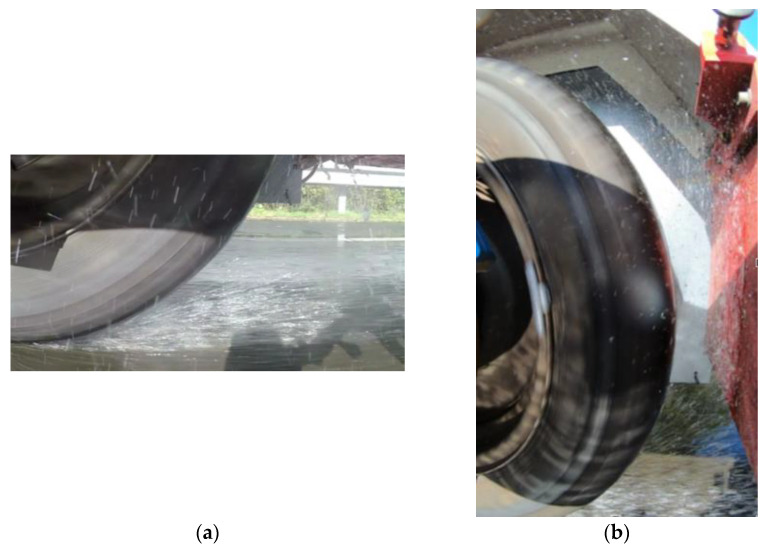
Water spray (**a**) view from camera 1; (**b**) view from camera 2 (videos were made without the PVC wheel arch to better visualize the water flows).

Illustrations of tire sprays are shown in Figure 9. Figure 9a clearly shows the torrent spray. The view from Figure 9b makes it possible to visualize both torrent and circumferential sprays.

After many unsuccessful (due to ambient light, background scenery, etc.) attempts to quantify the distribution of water droplets, it was decided to assess the intensity of water sprays by means of a subjective evaluation based on visual observations of the videos. Three intensity levels were defined based mainly on the size (visible, not visible) and the density of water droplets (Table 2).

From the proposed method, it was possible to estimate the intensity of water sprays as a function of surface macrotexture and vehicle speed. In Table 3, the wheel arch is divided into three parts which correspond to the field of vision covered by the three high-speed cameras (Figure 6). The colors indicate the following: the higher part of the wheel arch receives less water droplets than do the middle and the lower parts; and the intensity level is more scattered at the higher part compared to the two other parts of the wheel arch. The impact intensity increases as the speed increases. However, the effect of the surface macrotexture is not visible based on the surfaces tested in the present study.

The visualization of the water sprays shows that accelerometers located at the lower part of the wheel arch (A1 and A2, Figure 6) would provide more information than would the other accelerometers. Nevertheless, this choice, based on a subjective evaluation, required confirmation by results provided with accelerometric signals.

### 4.2. Analysis of Signals

Due to a limited number of connections of the data acquisition system, it was not possible to record data from the four accelerometers. Based on visual observation of water flows (low impacts on the higher part of the wheel arch), it was decided to exclude accelerometer A4 from the analyses.

#### 4.2.1. Raw Signals

**Figure 10:**
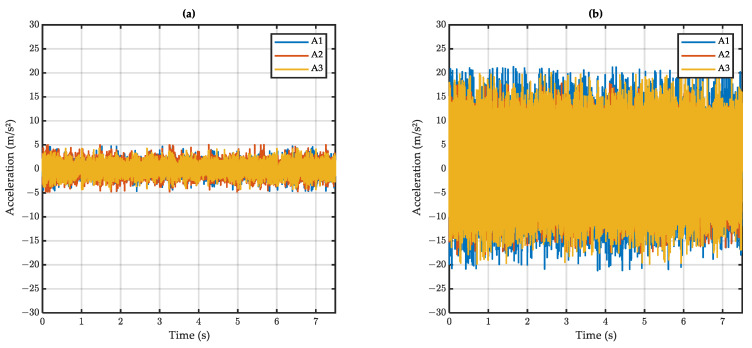
Raw accelerometric signal on (**a**) dry and (**b**) wet surface (surface C1, test speed 50 km/h).

The raw accelerometric signals at dry and wet conditions are presented in Figure 10a and Figure 10b respectively.

**Figure 11:**
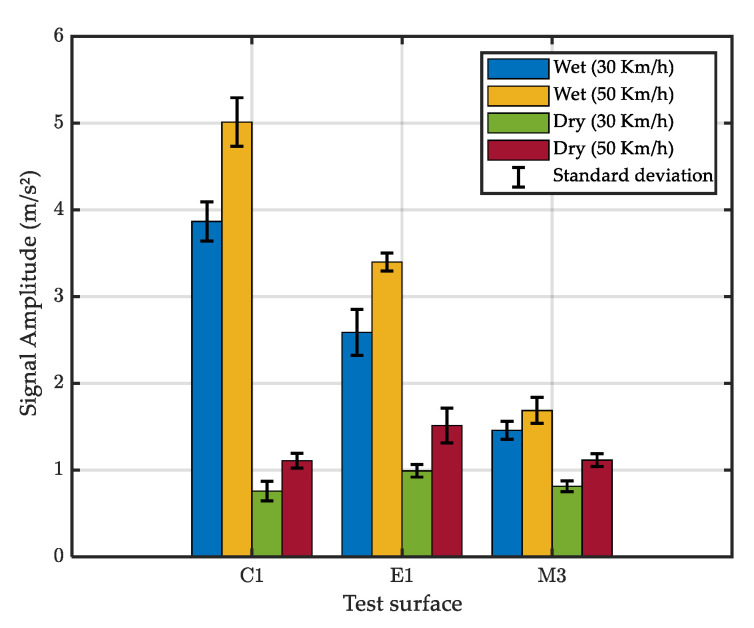
Mean amplitude of the raw signal (accelerometer A1).

The mean amplitude of accelerometric raw signals is presented in Figure 11. It can be seen that the wet amplitude is higher than the dry amplitude, meaning that the developed setup could differentiate between dry and wet surfaces. However, the difference between dry and wet was small on surface M3.

Considering the speed effect, it can be seen that a higher speed induces higher acceleration. The effect of the test surface, expressed in terms of MTD (see Section 3.3), is less clear: surface C1, with the lowest MTD, induces higher accelerations than do E1 and M3; however, the trend—low MTD inducing high accelerations—is not apparent for E1 and M3.

Despite the fact that the developed system could differentiate dry from wet surfaces, it was thought that the above results could be improved if noise—induced by the vehicle movement and other surface irregularities—was removed from the accelerometric signals. In the next section, a method of signal processing is proposed to remove noise and extract the relevant information from the raw signals.

#### 4.2.2. Filtered Signals

The recorded accelerations represent not only the impact of water droplets but also the effect of other factors that are not related to tire spray, such as vibrations due to the wheel suspension or the road surface irregularities. The aim of the signal processing presented in this section is to remove the effect of these secondary factors. This requires the knowledge of the properties of the signal noise, which is a difficult task because of the various origins of the noise.

**Figure 12:**
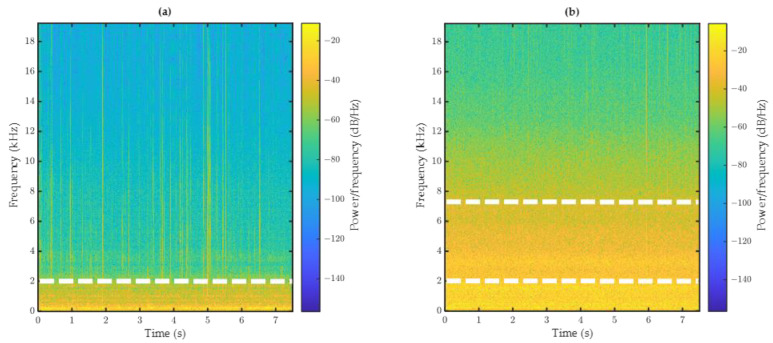
Spectrogram of (**a**) dry and (**b**) wet tests (surface C1, test speed 50 km/h).

To find the signature of the tire spray, a comparison between the raw signals in wet and dry conditions (at the same speed and on the same road surface) was performed. As an example, Figure 12 shows this comparison for tests performed at 50 km/h on surface C1. This figure represents the spectrogram of the signals obtained by the tests performed in dry and wet conditions. Spectrogram is a 3D presentation of the signal power (in color) for different frequencies (Y axis) during the sampling time (X axis). The signal power distribution in the frequency domain is presented by the power spectral density (PSD) and plotted in decibel scale for a better visualization.

The comparison of the two spectrograms (dry and wet) shows a clear difference in PSD value for frequencies higher than 2 kHz. In the dry condition, the PSD is lower than −60 dB/Hz for frequencies higher than 2 kHz, and in the wet condition, this is the case for frequencies higher than 7 kHz. This fact reveals that the effect of water droplet projections on the signal energy is mainly perceptible for frequencies between 2 kHz and 7 kHz.

**Figure 13:**
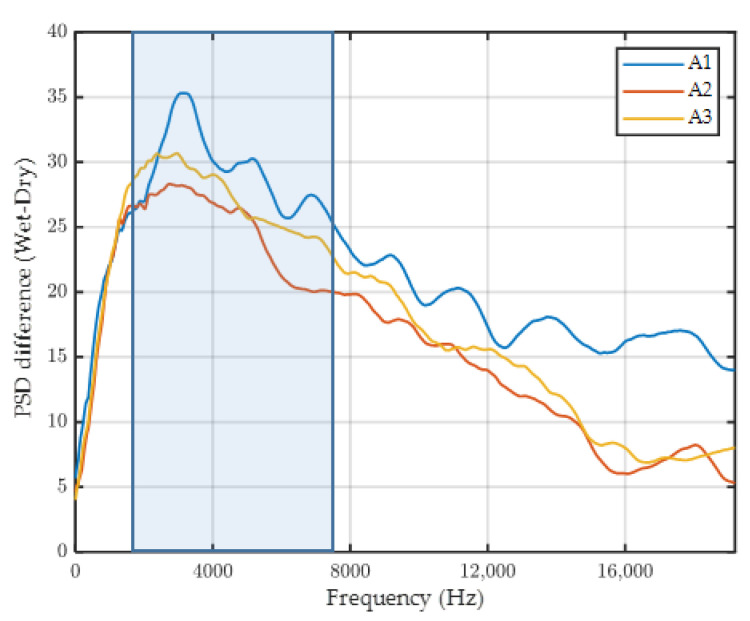
Difference of PSD between wet and dry signals (surface C1, test speed 50 km/h).

To better illustrate this fact, the PSD difference between wet and dry signals of the three accelerometers is presented in Figure 13. The difference curve shows a peak in the frequency zone between 2 kHz and 7 KHz (the blue box in Figure 13). In fact, in this zone, the difference between the dry and wet signal is the highest, and the effect of water droplet projection can be observed. It can be noticed that the peak is more pronounced for accelerometer A1; this result confirms the outcome of visual observations (lower-located accelerometers provide more relevant information) and helps to choose the appropriate location—A1—for the accelerometers (the first need expressed in the introduction).

**Figure 14:**
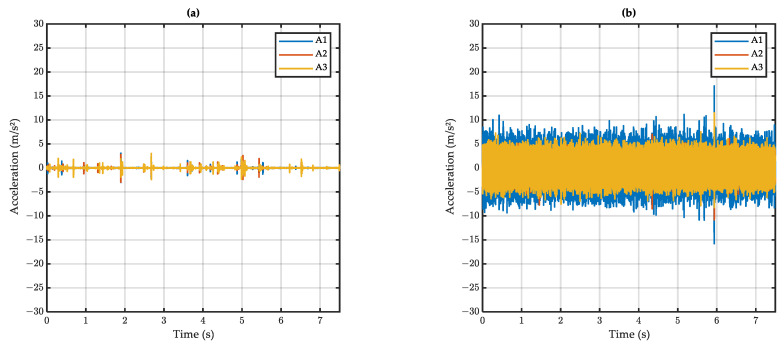
Filtered signals for (**a**) dry and (**b**) wet tests (surface C1, test speed 50 km/h).

Based on the above results, a Chebyshev filter was used with a passing band of 2 kHz to 7 kHz to filter the raw signals. Results showed that the filter reduced the observed noise for the tests performed in the dry condition (Figure 14a) and, as a consequence, the filtered signal in Figure 14b should reflect only the effect of water droplets.

**Figure 15:**
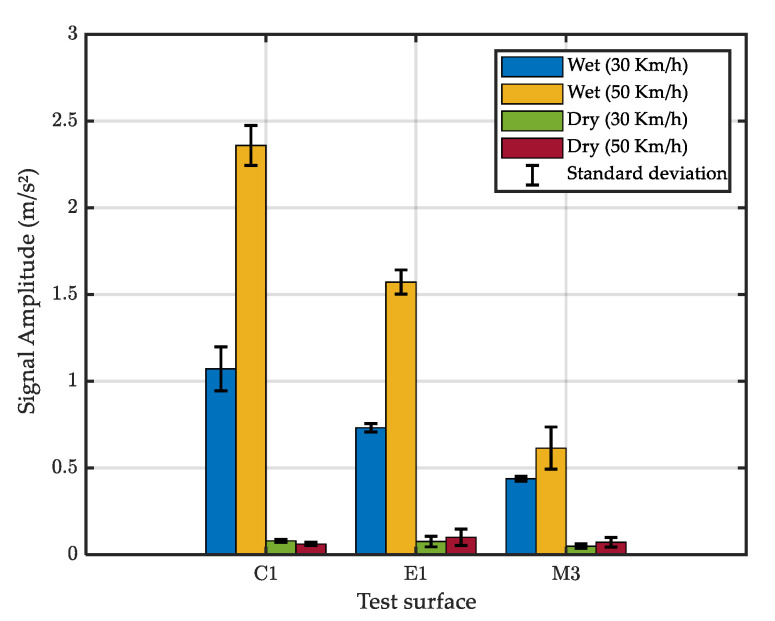
Mean amplitude of the filtered signal.

Figure 15 shows the mean amplitude of the filtered signals for the test surfaces. Comparing this figure with Figure 11 shows that the proposed filter removed noise in the dry tests and better emphasized the effect of water spray.

**Figure 16:**
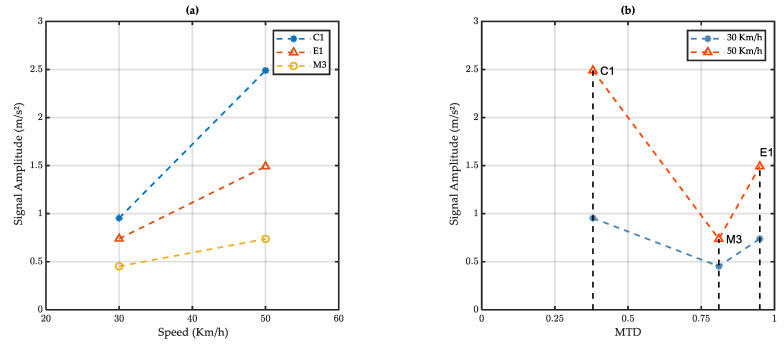
Mean signal amplitude of the filtered signal for wet tests as a function of (**a**) vehicle speed and (**b**) road surface macrotexture.

The effect of the test speed on the signal amplitude for tests performed in the wet condition can be seen in Figure 16a. It can be seen that the signal amplitude increases with the test speed for the three tested surfaces but the increase rate depends on the test surface.

The variation of the signal amplitude with the surface MTD presented in Figure 16b shows that the link between these two parameters is not obvious. Before going further in terms of discussions, it is necessary to check the variation of actual water depths with the vehicle speed and the surface texture.

### 4.3. Estimation of Water Depths

#### 4.3.1. Analysis of Measured Water Depths

**Figure 17:**
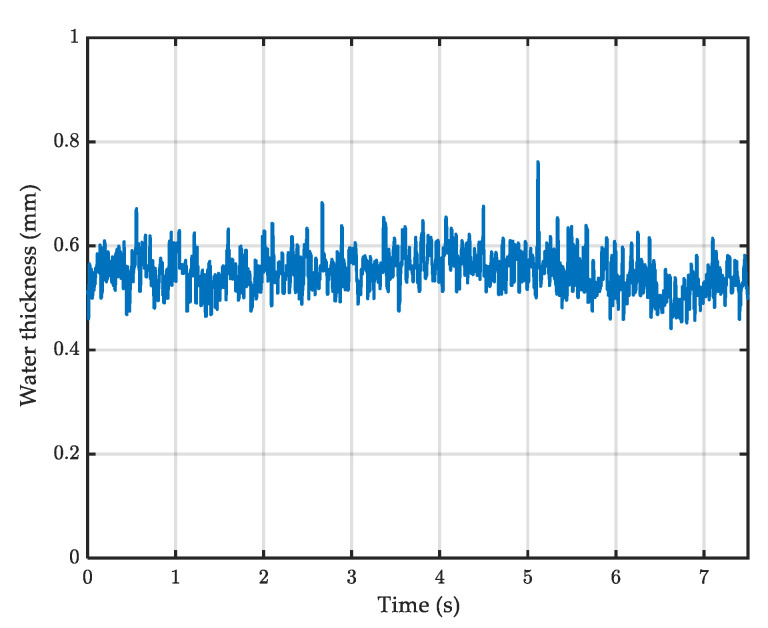
Example of measured water depth (surface C1, test speed 50 km/h).

Figure 17 shows an example of the water depth measured by the Aquasens sensor (surface C1, test speed 50 km/h).

**Figure 18:**
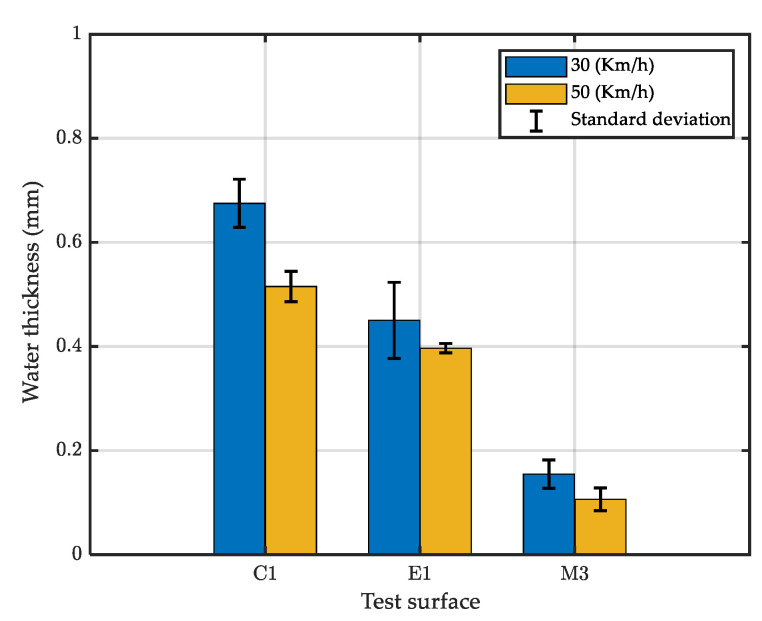
Mean value of water depths measured by the Aquasens sensor.

The water depth for each test is calculated by averaging values recorded on the defined test length (70 m). It can be seen from Figure 18 that, for the same surface, the measured water depth was lower for tests performed at higher speed. This surprising result can be explained by the fact that when the vehicle speed increases, the travelling time decreases (the test length is fixed), and so less water is spread on the surface (because the water flow is fixed).

Results showed that the surface texture affected the recorded water depth: surfaces C1 and M3 had the highest and the lowest water thicknesses, respectively. This result is logical because C1 and M3 are qualified as “close” and “open” surfaces, respectively. However, it can be seen that the MTD parameter cannot entirely explain the variation of water depths; otherwise, the water depth on surface M3 (MTD = 0.85) should be higher than that on surface E1 (MTD = 0.95). Actually, as surface M3 is a very thin asphalt concrete, it is more porous than surface E1. As surface M3 can store more water than can surface E1, for the same quantity of water spread on the surface, the water depth is lower on surface M3 than on surface E1.

While more extensive tests are needed to confirm the relevance of the above discussion, it can already be said that the mean texture depth is not enough to characterize a road surface with respect to its wetness. Other parameters, such as the void percentage or the void volumes (which can be calculated from a cumulative height distribution curve, also known as Abbott curve), would provide additional information and help to better understand the link between surface texture and water depth.

#### 4.3.2. Relationship between Accelerations and Water Depths

**Figure 19:**
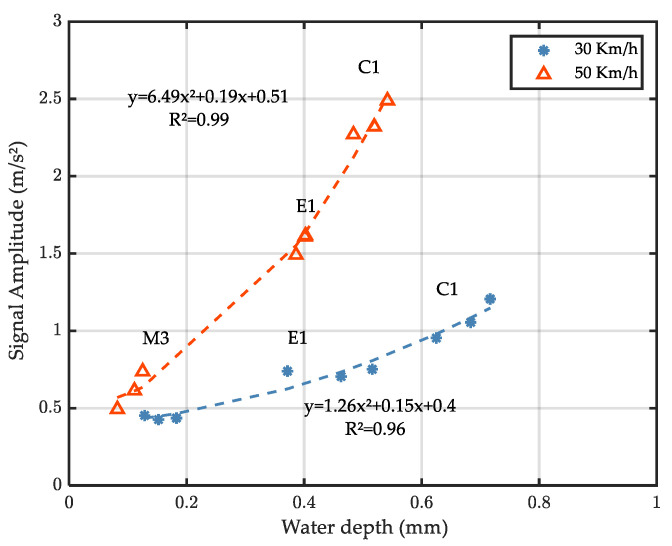
Evolution of the filtered signal amplitude with water depth.

Figure 19 shows the relationship between the mean amplitude of the filtered signals and the measured water depth for accelerometer A1. The dotted lines are obtained by fitting the experimental data by a 2nd degree polynomial function.

Results show that, for each test speed, a tight relationship can be established between the water depth and the mean amplitude of the filtered signal regardless of the road surfaces. While further tests are needed to validate these first results and determine the link between the parameters of the fitting function and the test speed, the existence of a master curve for all surfaces and the high value of the coefficient of correlation (r2) are promising in view of estimating water depths from the developed setup.

It can be noticed that this graph is not in contradiction with results in Figure 15 (higher speed induces higher accelerations) and Figure 18 (higher speed induces lower water depth). The consequence of these trends is that the response of the accelerometer seems to be more sensitive to variations of water depths when the vehicle speed increases: for example, when the water depth increases from 0.2 mm to 0.4 mm, the acceleration difference is 0.25 mm/s^2^ (0.5 to 0.75) at 30 km/h and 1 mm/s^2^ (0.5 to 1.5) at 50 km/h. This sensitivity can be useful for driver warning because skid resistance loss—and thus the accident risk—is more important when the vehicle speed is high on wet roads.

## 5. Conclusions

This work was conducted to evaluate the feasibility of using accelerometers as an indirect mean to estimate water depths on road surfaces. The idea is to measure vibrations induced by the impact of water droplets, projected by the rolling wheel, on the front wheel’s arch liner and establish the link between accelerations, induced by the wheel arch vibration, and water depths. As a first approach, a trailer equipped with a wheel and towed by a van was used because of its dynamics, which is representative of a quarter of a passenger car, and the possibility to visualize water flows by means of high-speed cameras.

The first objective of the study is to choose a suitable location for the accelerometers. Four locations were tested. From visualization of water flows (mainly torrent and circumferential sprays), the best place is the lower part of the wheel arch—near the road surface—where the intensity of water sprays (evaluated subjectively) is the most important. Signal analysis confirmed this choice by showing a more marked feature—peak—on the signal provided by the accelerometer located at this place compared with the other accelerometers.

The second objective of the study is to develop a protocol for signal processing. A Chebyshev filter was used with a passing band of 2 kHz to 7 kHz to filter the raw signals and remove noises induced by vehicle movements, surface irregularities, etc. The filtered signals make it possible to obtain a smooth signal in dry condition; therefore, signals recorded under wet conditions should reflect only the effect of water droplets.

The third objective of the study is to determine a relationship between accelerometric signals and actual water depths. With the mean amplitude of the filtered signals being used, results show that the mean amplitude increases with increasing water depth and increasing speed. Based on the test program conducted in this study (three surfaces, two speeds), a master curve relating the signals’ mean amplitude to the water depth can be obtained regardless of the road surfaces and can be modelled by a quadratic function. The mean acceleration amplitude–water depth curve is speed dependent: higher speed induces a steeper curve, meaning that the accelerometers are more sensitive to variations of water depths when the speed increases.

The main finding of this study, compared with previous ones, is the establishment of a clear relationship between the measured accelerations and the water depths. The next step of the study is the implementation of the system in a passenger car (front wheel arch) and testing with more exhaustive configurations in terms of road surfaces and vehicle speeds. The development of an on-board friction estimator forms the final aim of this research and is part of future investigations.

## Figures and Tables

**Table 1:** Test surfaces.

Surface	Texture	MTD (mm)
C1	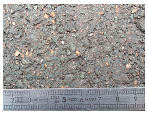	0.38
E1	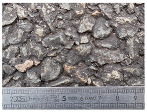	0.95
M3	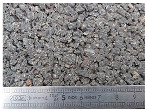	0.85

**Table 2:** Subjective evaluation of the intensity of water sprays.

	Intensity Level		
	Low	Medium	High
Illustration (photos extracted from videos)	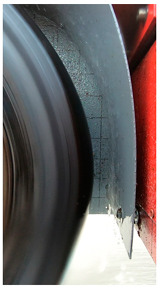	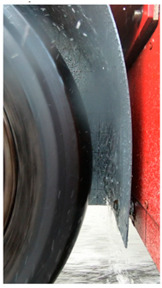	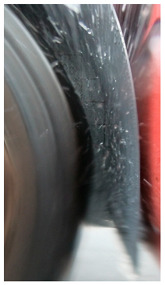
Description	Droplets barely visible on part of or the whole wheel arch	Droplets visible on the whole wheel arch	Big droplets densely distributed on the whole wheel arch

**Table 3:** Intensity of water sprays as a function of surface macrotexture and vehicle speed.

Surface	C1			E1		
Run	1	2	3	1	2	3
30 km/h	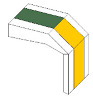	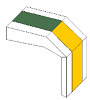	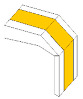	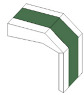	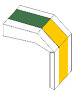	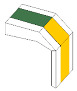
50 km/h	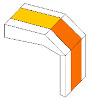	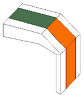	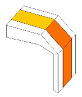	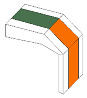	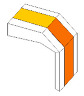	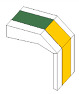

## Data Availability

The data presented in this study are available on request from the corresponding author. The data are not publicly available due to a confidentiality agreement within the framework of the ENA project.
